# WikiPathways: capturing the full diversity of pathway knowledge

**DOI:** 10.1093/nar/gkv1024

**Published:** 2015-10-19

**Authors:** Martina Kutmon, Anders Riutta, Nuno Nunes, Kristina Hanspers, Egon L. Willighagen, Anwesha Bohler, Jonathan Mélius, Andra Waagmeester, Sravanthi R. Sinha, Ryan Miller, Susan L. Coort, Elisa Cirillo, Bart Smeets, Chris T. Evelo, Alexander R. Pico

**Affiliations:** 1 Department of Bioinformatics - BiGCaT, NUTRIM, Maastricht University, Maastricht, 6229 ER Maastricht, The Netherlands; 2 Maastricht Centre for Systems Biology (MaCSBio), Maastricht University, Maastricht, 6229 ER Maastricht, The Netherlands; 3 Gladstone Institutes, San Francisco, California, CA 94158, USA; 4 Micelio, Antwerp, 2180 Antwerp, Belgium; 5 Keshav Memorial Institute of Technology, Hyderabad, Telangana 500029, India

## Abstract

WikiPathways (http://www.wikipathways.org) is an open, collaborative platform for capturing and disseminating models of biological pathways for data visualization and analysis. Since our last NAR update, 4 years ago, WikiPathways has experienced massive growth in content, which continues to be contributed by hundreds of individuals each year. New aspects of the diversity and depth of the collected pathways are described from the perspective of researchers interested in using pathway information in their studies. We provide updates on extensions and services to support pathway analysis and visualization via popular standalone tools, i.e. PathVisio and Cytoscape, web applications and common programming environments. We introduce the Quick Edit feature for pathway authors and curators, in addition to new means of publishing pathways and maintaining custom pathway collections to serve specific research topics and communities. In addition to the latest milestones in our pathway collection and curation effort, we also highlight the latest means to access the content as publishable figures, as standard data files, and as linked data, including bulk and programmatic access.

## INTRODUCTION

**Figure 1.**
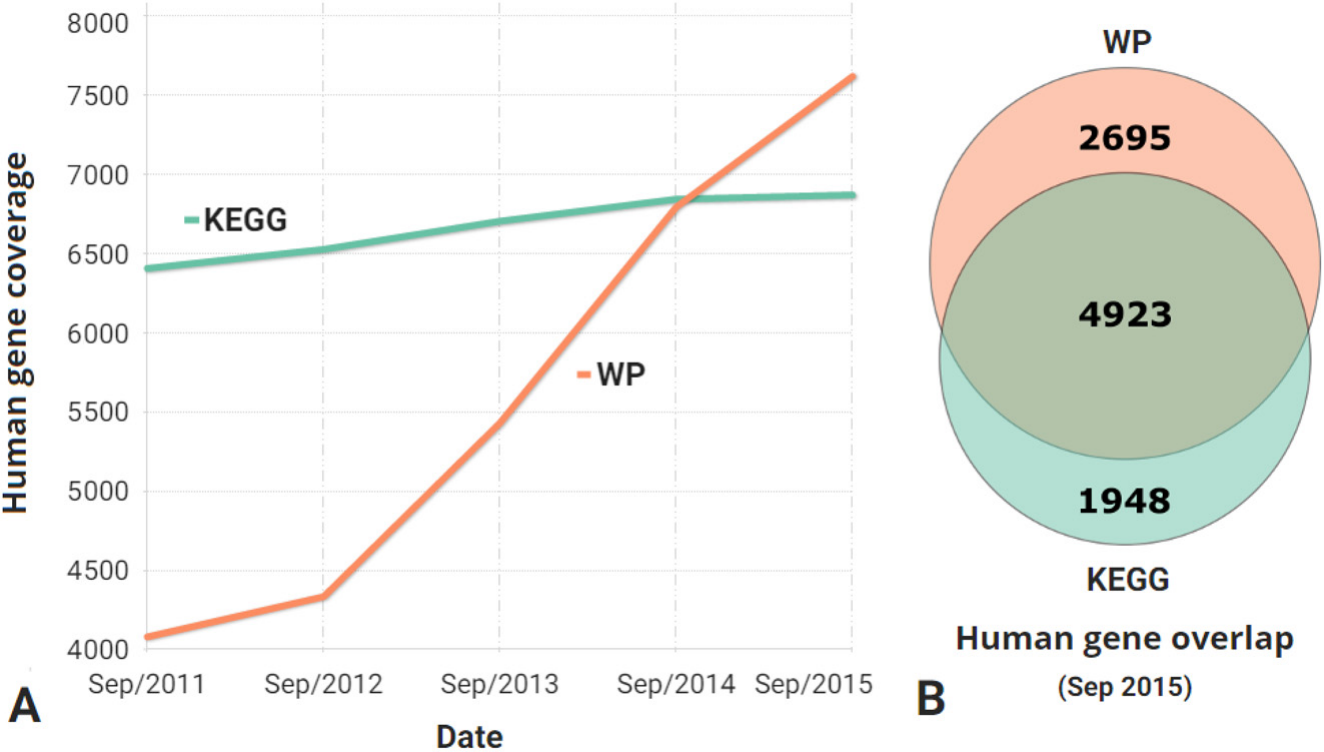
**Human gene coverage in WikiPathways**. (**A**) Taking KEGG as the gold standard for pathway databases, we plot the growth of WikiPathway (WP) coverage over the past 4 years. WikiPathways shows a relative trend of higher growth. Last year the absolute coverage matched that of KEGG and continues to climb. (**B**) In terms of gene-for-gene coverage, WikiPathways covers the bulk of the content found in KEGG's canonical pathways (overlap). The proportional Venn diagram also shows that a third of our content is unique. WikiPathways and KEGG unique human gene counts were made by extracting identifiers from archived databases and unifying to Ensembl release 80.

Launched in 2008 as an experiment to see if crowdsourcing could work for a pathway archive, WikiPathways started out with 500 pathways across six species maintained by four individuals (1). Today, WikiPathways (http://www.wikipathways.org) contains over 2300 pathways across over 25 different species. The human pathway collection is the largest and most active collection by species, having increased 6-fold to include 640 pathways. In terms of coverage of unique human genes, WikiPathways is comparable to KEGG (2) (Figure 1). Our advantage going forward lies in our scalable, community-based curation and unrestricted pathway model, accepting any pathway that researchers find useful in their work. Over a recent 12-month period, 208 individuals made over 3200 edits to 1048 pathways at WikiPathways. This level of activity simply cannot be matched by internal teams of hired curators. Consider that over the same period, only 16 KEGG pathways were updated (http://www.kegg.jp/kegg/docs/upd_map.html). The quantity, quality and diversity of content in WikiPathways is due to hundreds of individuals contributing their time and domain knowledge each year.

It is not enough, however, to only amass a large collection of pathways; it must also be actively distributed and made maximally accessible. WikiPathways guarantees free and open access to its full collection under a Creative Commons (CC BY 3.0) license and liberal terms of use ensuring that content contributed by the community will always be available to the community (http://www.wikipathways.org/index.php/WikiPathways:License_Terms). This extends to individual and bulk downloads of all available formats, as well as all means of programmatic access. Furthermore, as an open source collaboration from the start, the technical development of the WikiPathways platform itself is also open to community participation (https://github.com/wikipathways).

In the following sections, we highlight updates at WikiPathways that are relevant across the spectrum of researchers, from bench biologists to computational biologists. We begin with the most recent additions to the breadth and depth of content at WikiPathways, including website and software updates that make this content easy to find and use in data analysis and visualization. In the next section, we focus on new tools available to pathway authors and curators, and new avenues to publishing pathways and organizing communities of researchers around shared pathway models. We conclude with the latest updates for data scientists and programmers interested in our new data formats, web service methods, linked data and embed code. Each section includes its own description of future plans pertaining to its topics.

## UPDATES FOR BIOLOGISTS AND CHEMISTS

WikiPathways was created by research groups with active transcriptomics and metabolomics projects in order to support high-throughput data analysis and visualization (1,3). Providing a pathway resource that scales with ever-expanding research activity remains a primary aim of WikiPathways. The updates described in this section are targeted to those who might use pathway models in their own research programs.

### Diversity and depth of new pathways

Building upon the canonical set of pathways found at most pathway archives, we have made a concerted effort to also capture more specialized models of biology. Our most successful approach has been to engage established research communities already focused on a model organism, class of pathway or particular cell type. For example, the curators at WormBase launched a dedicated portal at WikiPathways for diverse pathways related to *C. elegans* as a model organism (4). The WormBase team has added and refined over a dozen pathways that are highlighted as ‘WormBase Approved’ and are available for viewing, download, and further editing like any other content at WikiPathways. Our two most recent collaborations are with research consortia to model the latest research in stem cell biology (Progenitor Cell Biology Consortium, progenitorcells.org) and extracellular RNA (Extracellular RNA Communication Program, exrna.org). The exRNA portal at WikiPathways has accumulated over 45 pathways in the last year that highlight exRNA and miRNA roles in everything from differentiation and inflammation to ovarian cancer and Alzheimer's (http://www.wikipathways.org/index.php/Portal:ExRNA/FeaturedPathways). We coordinate with the exRNA community to make sure every consortium publication that includes a pathway figure is also captured as a properly modeled pathway.

Since our last update, we have established a collaboration with Reactome to boost the human pathway collection at WikiPathways. Reactome shares our goal of providing a free, open access collection of peer-reviewed pathways and associated software tools (5). With a more centralized curation process, Reactome can benefit from having access to more open, community curation at WikiPathways, in addition to having their content distributed in additional formats, services and analysis tools. Likewise, WikiPathways benefits by hosting their uniform pathway models that are compliant with systems biology standards such as those described at http://www.sbgn.org (6) and http://www.sbml.org (7). This collaboration has resulted in the recent conversion and inclusion of more than 300 human pathway models into our collection (http://www.wikipathways.org/index.php/Portal:Reactome).

In addition to crowdsourced curation, watchlist activity and curation tag procedures, we have instituted another level of quality control at WikiPathways in the form of a roster-based curation protocol. Year-round, on any given week, there is a designated administrative curator who executes an 8-step quality control protocol encompassing the entire pathway archive (http://www.wikipathways.org/index.php/Help:Curation_Protocol). The protocol steps include screening all new edits, orienting new users, fixing poorly annotated pathways and molecules, as well as assessing the competence of content for common downstream data analysis and visualization workflows (see the next section). Part of this process is computer-assisted by validation reports from regular run bots and tests. Anyone with a demonstrated interest in WikiPathways can apply to join the administrative curation roster.

With a WikiPathways user account, you can manage your own ‘Watchlist’ of pathways to be notified of changes. However, you can also track general updates to the project and content via Twitter (@WikiPathways), Tumblr (http://www.wikipathways.tumblr.com), and Google Group (wikipathways-discuss).

### Utility of pathways to researchers

Pathway models have proven themselves immensely useful for computational analysis and interpretation of large-scale experimental data. Ideally, those models can be used directly within commonly used pathway and network analysis tools to increase the usability for researchers. Therefore, we developed extensions for the pathway analysis tool PathVisio (8) and the network analysis tool Cytoscape (9) to make the integration of WikiPathways pathways in the analysis of biological data as easy as possible.

PathVisio is a commonly used pathway editor, visualization and analysis tool, and additional features are provided via the plugin repository. The WikiPathways plugin was developed to support browsing and searching the database directly from within PathVisio (http://www.pathvisio.org/plugin/wikipathways-plugin). The plugin also allows users to create and upload new pathways, or edit and curate existing pathways. Within PathVisio, researchers have access to an array of core and plugin features to support the analysis of omics data and customized data visualization.

The abstract representation of pathways as networks of only nodes and edges are ideal for topological analyses, network extension, network visualization and other advanced network analysis methods. We developed the WikiPathways app for the widely adopted network analysis and visualization software Cytoscape to enable researchers to use WikiPathways pathways in network analysis approaches (10). The app supports searching and loading pathways from WikiPathways through ‘Online database import’, or importing a pathway from a local GPML (Graphical Pathway Markup Language) file. Furthermore, the app provides two ways to load pathways: as an annotated pathway ideal for data visualization and as an abstract network ideal for graph analysis.

### Overview of interface updates

Besides extending the pathway content and developing new analysis tools, we are also focusing on updating our interface to provide a user-friendly and intuitive website. New video tutorials highlight the latest navigation and editing features, and a new interactive pathway viewer for tissue-specific expression data has been added.

Recently, our help documentation was updated and new video guides were made available, including ‘tours’ through WikiPathways interfaces. The ‘Navigation Tour’ quickly highlights the search, browse and download options, as well as the rich content found on an example pathway page (see Supplementary Data: Navigation Tour). The ‘Editing Tour’ video provides an overview of various means of editing a pathway, including the new Quick Edit option, the full-featured Applet option, and the offline-capable PathVisio options that leverages the new WikiPathways plugin (see Supplementary Data: Editing Tour).

**Figure 2.**
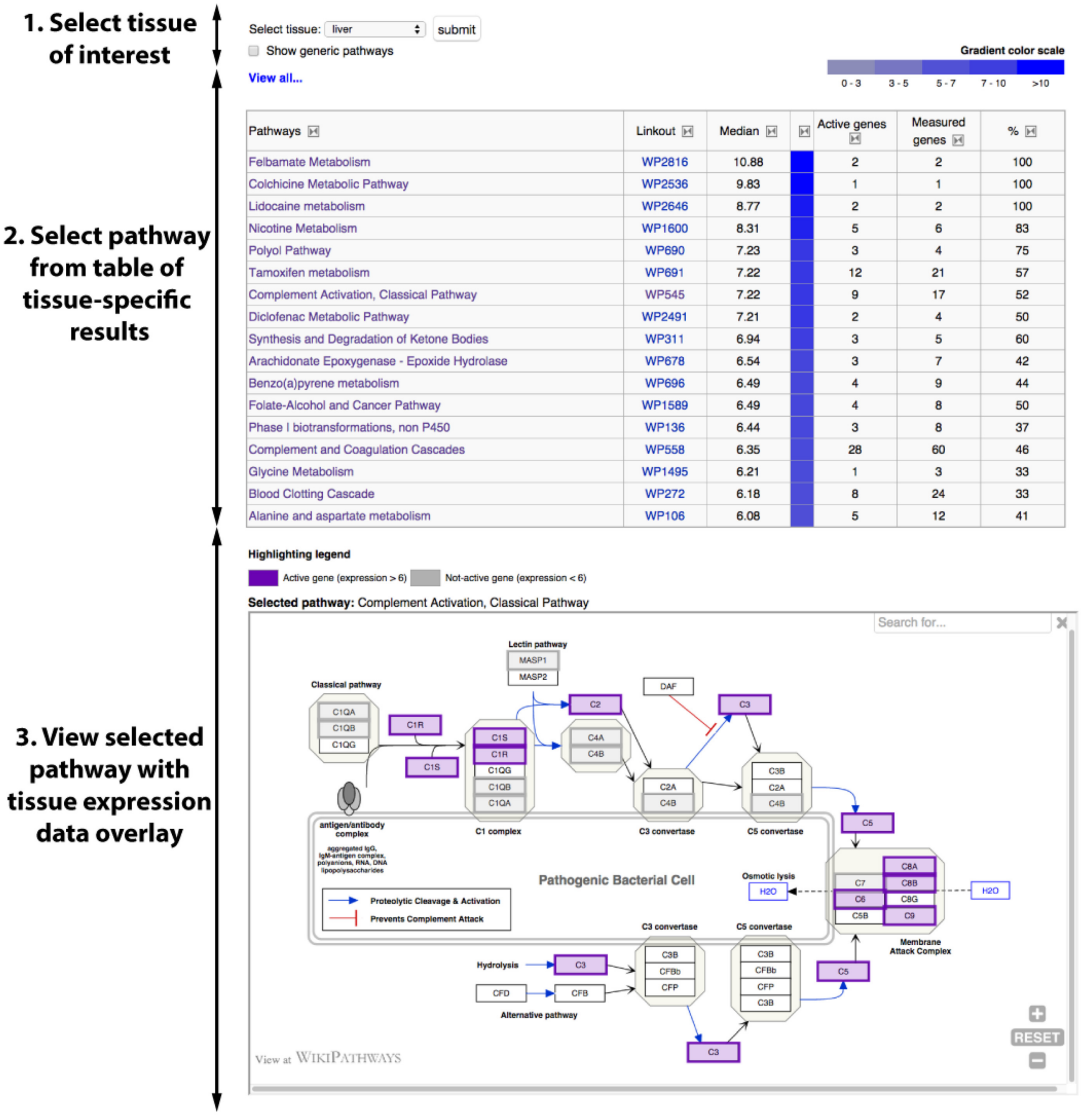
**TissueAnalyzer**. Select one of ∼30 tissues from the pulldown list at the top of the page. TissueAnalyzer will then generate a table of pathways based on their selective overrepresentation of active genes in the selected tissue. Select a pathway from the table to open an interactive, web-embedded view with a tissue expression data overlay.

In one of the latest releases, we introduced TissueAnalyzer (http://www.wikipathways.org/index.php/Special:TissueAnalyzer). The goal of this feature is to combine the pathway knowledge from WikiPathways with baseline expression data for 27 different tissues from Expression Atlas (11,12) to study the activity of biological pathways in a specific tissue. TissueAnalyzer provides a ranked table of pathways per tissue based on their median expression and the active genes in each pathway (Figure 2). The interactive pathway viewer shows selected pathway diagrams and highlights the active genes, so researchers can immediately see which genes in the pathways are expressed in the selected tissue.

### Future plans and community channels

For researchers navigating and consulting WikiPathways, we will be adding *infoboxes* to each pathway page, providing summary information about the pathway, its contents and how it relates to other pathways and public data, such as the TissueAnalyzer results described above. The goal of the infobox will be to provide context and connections based on underlying analytics to better inform researchers as to the relevance of a given pathway to their work.

We will continue to engage research communities and consortia as they come online and present a need for pathway modeling and analysis. We encourage any existing communities with such a need to contact the authors and get involved. This will be an ongoing effort to capture and distribute new pathway models that include new molecular types, interactions and associations, as our collective knowledge continues to diversify and deepen.

## UPDATES FOR PATHWAY AUTHORS AND CURATORS

There is a thin line between a user and a contributor at WikiPathways. The updates described in this section highlight new editing and publishing tools to enable researchers to seamlessly switch between consuming and contributing. Casual and full-time curators alike can model, annotate and correct pathways of interest for immediate distribution through multiple outlets.

### New curation tools

**Figure 3.**
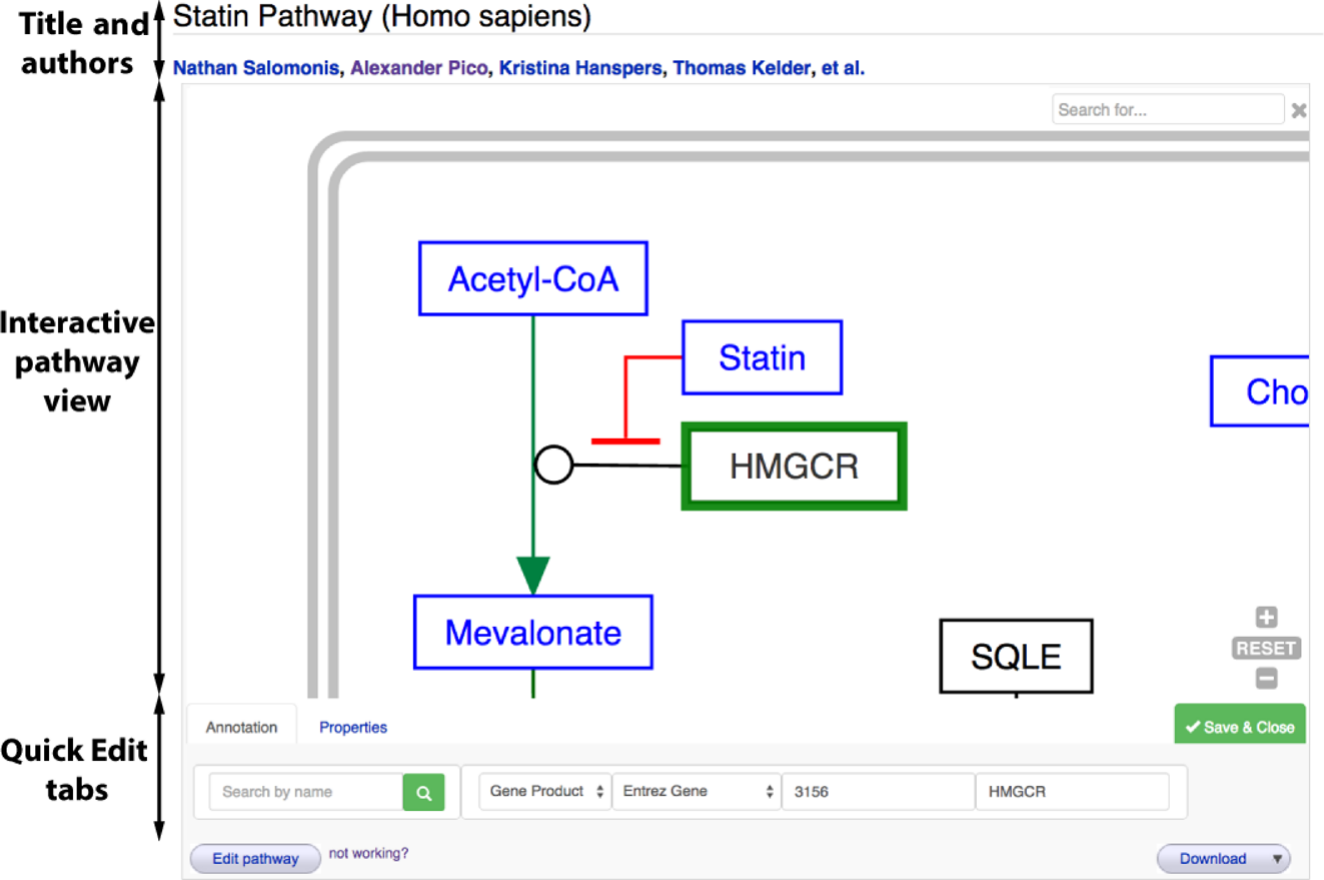
**The new Quick Edit feature**. At the base of the interactive pathway view, a series of quick access tabs allow editing of DataNode annotations and properties. The annotation tab provides a DataNode search feature, plus manual override options for type, data source, identifier and label. The properties tab (not active here) provides control of DataNode color and label font attributes. The button on the far right will save any changes made and close the Quick Edit set of tabs.

We have developed a number of new tools to make pathway editing as easy as possible, so everyone can contribute their specialized knowledge. The most radical approach we introduced this past year is the *Quick Edit* feature. Activated by clicking the Quick Edit button, the tabbed interface allows you to directly edit the pathway model within the existing interactive display window (Figure 3). These features are built upon our new pvjs technology (https://github.com/wikipathways/pvjs), a JavaScript solution that works immediately in all modern browsers, eliminating the need to install additional software. The first set of Quick Edit tabs support the editing of DataNode annotations, labels, fonts and colors.

For full-featured editing capability, including offline support, we developed the WikiPathways plugin for PathVisio. The plugin allows you to use your WikiPathways username and password to query, import, and upload pathways to and from the site. Together with other curation-focused plugins, the WikiPathways plugin facilitates the curation of WikiPathways pathways. The ‘Editing Tour’ video includes the latest updates to the WikiPathways plugin for PathVisio and the Quick Edit feature (see Supplemental Data: Editing Tour).

A key aspect of modeling pathways is assigning standardized identifiers to its components. This allows molecules, for example, to not only be generally recognizable, but also to be associated and visually overlaid with data. In addition to molecules and pathways, we recently added support for labeling interactions and complexes with standard identifiers as well.

### Publishing pathways

At the top of each pathway page is a dynamic author list, acknowledging the original author (always in the first position) and all subsequent authors in order of the number of edits made. For every edit, a version of the pathway is stored indefinitely as a uniquely citable reference (http://www.wikipathways.org/index.php/How_to_cite_WikiPathways). In turn, each pathway has its own bibliography. Pathways from WikiPathways can thus be used both as figures in papers and as references, like collaboratively written review articles. Every article that properly cites WikiPathways is automatically added to our weekly Tumblr feed (http://www.wikipathways.tumblr.com). We also established automatic distribution channels to diverse third-party resources where researchers might find pathways associated with Entrez Gene records (NCBI Biosystems (13)), other BioPAX-formatted pathways (PathwayCommons (14)), microRNA target information (miRWalk2.0 (15)), and drug-discovery data (Open PHACTS (16)).

Authors now have the option to link their WikiPathways account with their Open Researcher and Contributor ID (ORCID) (17), using a template contributed by Andy Mabbett (http://www.wikipathways.org/index.php/Template:User_ORCID). The template adds an ORCID badge and category to their WikiPathways profile page, enabling programmatic access to their contributions as well.

### Supporting communities

WikiPathways is a community curated database and we work closely with different research communities to support them in modeling their pathways of interest. Additionally, we provide the capability for communities to host their own portal sites to highlight any subset of pathways relevant to their community. Newer portals at WikiPathways include ones for WormBase, exRNA research, human disease models and plant biology (18). We continue to encourage research communities and consortia with an interest in using and modeling biological pathways to consider the opportunity to create their own portal site to spotlight their community at WikiPathways.

### Future plans

In the coming months, we will be adding new tabs to the Quick Edit feature to support all common editing activities. In parallel, we will build additional crowdsourced—and even gamified—options for compiling new pathway information, e.g. by parsing pathway figures from articles. In an effort to enhance the use of WikiPathways as a publishing tool, we will add sharing and citation links to the coming *infobox* feature. We will also be working on an interactive export option with preset graphics, or skins, to produce professional and compelling figures for publications and presentations. In parallel with these enhancements, we will be working with publishers to provide interactive views of pathway diagrams in addition to static figures, as supplemental material embedded into their online article platforms (see Supplementary Data: Interactive PathwayWidget).

## UPDATES FOR DATA MINERS AND PROGRAMMERS

WikiPathways content is most effective when utilized as a collection. We provide multiple standardized formats and methods to programmatically query and model our pathway content for downstream analysis and visualization. With the latest updates, content can be pulled into various scripting environments, pipelines, standalone applications, and a myriad of web-centric workflows and interactive displays.

### Data formats and availability

WikiPathways pathways and collections can be downloaded in several different machine readable formats. Moreover, the pathway diagrams can be downloaded as high quality figures for publication in PNG, SVG and PDF formats. The curated collections for all supported species can be downloaded from http://www.wikipathways.org/index.php/Download_Pathways. We also routinely convert our content into the community-driven pathway exchange format, BioPAX (19). One of the best ways to access our BioPAX content is via Pathway Commons (14), which validates and compiles pathway models from many resources.

WikiPathways maintains its own web service for programmatic access, promoting the integration of biological pathways in existing pipelines and applications (20). The web service provides functions to list all available organisms and pathways, search for pathways in which a specific molecule is present, retrieve a list of all molecules in the pathway or get detailed information about a specific pathway including the GPML file. Recently, a new web service function was added to retrieve all contributions of a user by providing a username or ORCID. A complete list of web service functions can be found at http://webservice.wikipathways.org. Thousands of web service calls per month show the importance of WikiPathways content in biological data analysis workflows. The web service returns XML by default, but it can also return JSON to support the active community of developers of web-based visualization and analysis. JSON has become a preferred format because it is readily parsed into data structures and is more compact than alternatives such as XML. Semantic context is added to returned pathway information by means of JSON-LD (http://json-ld.org), providing linked data support, while maintaining the lightweight and flexible qualities of the JSON format.

We also publish WikiPathways content on the semantic web, where it can be queried through SPARQL at http://sparql.wikipathways.org or the Open PHACTS API (16). Integrated into the semantic web as unified resource identifiers (URIs), our content is being used to link pathways to concepts such as disease genes curated by DisGeNET (21). On the website users can find some general information about this effort, including over 50 example queries (http://www.wikipathways.org/index.php/Help:WikiPathways_RDF).

### Data analysis and visualization

Pathways contain a lot of information about biomolecules and their interactions that provides valuable context and fodder for data analysis and visualization. We provide several different ways to programmatically access and use WikiPathways content. The WikiPathways plugin for PathVisio and the WikiPathways app for Cytoscape, as previously described, are examples of powerful standalone applications—each with their own extensible and scriptable potential—that have direct access to WikiPathways and perform a wide variety of analytical and visualization functions. The next two examples are targeted to programmers seeking to integrate pathway visualizations into their web projects or to integrate pathway analysis into their automated workflows.

Custom, interactive pathway visualizations can be integrated into websites by means of our PathwayWidget (http://www.wikipathways.org/index.php/PathwayWidget). By embedding a short HTML code snippet, a specified pathway will be displayed in an interactive pathway viewer from WikiPathways, allowing pan and zoom, search and annotation panel popups. The PathwayWidget syntax also supports specifying a list of pathway elements and a corresponding list of colors to highlight, for example, specific genes and color them based on data values or any other criteria relevant to the site (see Supplementary Data: Interactive PathwayWidget).

A new programmatic interface for PathVisio called PathVisioRPC was recently introduced (22). It provides an API for pathway editing, data visualization and pathway statistics through an XML-RPC interface to all major programming languages, including R, Perl, Python, Java, C, C++ and PHP. Especially for larger analyses, the automation of repetitive tasks can save a lot of time; it also makes analyses more reproducible. Furthermore, it allows researchers to integrate pathway visualization and statistics into their data analysis workflows.

### Future plans

We will continue to develop new features for the standalone PathVisio and Cytoscape applications to take advantage of the expanding content at WikiPathways. However, we are also dedicated to providing web-centric resources, widgets and tools for other developers to leverage and incorporate in their own projects. We plan to couple the PathwayWidget with our web services to provide simplified, single-purpose versions of the widget to make it even easier, for example, to deploy interactive pathway views starting with arbitrary gene names, ontology terms or keywords. Our linked data formats introduced here will also see further development as we identify new and meaningful semantics to associate with our biological pathway models.

## CONCLUSION

The WikiPathways project is essentially comprised of an open source development team, an open access, collaborative platform and, most importantly, a diverse community of contributors. The updates presented here demonstrate the success of our approach so far and the active support for researchers, curators and tool developers. We welcome all potential collaborators interested in collecting, curating and using pathway content, as well as in the development of new software tools and services. Overall, we report tremendous growth in content and features since the last update (3), and a continued commitment to capture every pathway of interest and disseminate it in as many useful ways as possible.

## AVAILABILITY

1. WikiPathways: http://www.wikipathways.org
2. Download: http://www.wikipathways.org/index.php/Download_Pathways

## SUPPLEMENTARY DATA

Supplementary Data are available at NAR online.
